# Presentation and integration of multiple signals that modulate oligodendrocyte lineage progression and myelination

**DOI:** 10.3389/fncel.2022.1041853

**Published:** 2022-11-14

**Authors:** Christopher D. Fekete, Akiko Nishiyama

**Affiliations:** Department of Physiology and Neurobiology, University of Connecticut, Storrs, CT, United States

**Keywords:** oligodendrocyte, OPC, NG2, neuron-glia communication, trafficking, Fyn, BDNF, L1

## Abstract

Myelination is critical for fast saltatory conduction of action potentials. Recent studies have revealed that myelin is not a static structure as previously considered but continues to be made and remodeled throughout adulthood in tune with the network requirement. Synthesis of new myelin requires turning on the switch in oligodendrocytes (OL) to initiate the myelination program that includes synthesis and transport of macromolecules needed for myelin production as well as the metabolic and other cellular functions needed to support this process. A significant amount of information is available regarding the individual intrinsic and extrinsic signals that promote OL commitment, expansion, terminal differentiation, and myelination. However, it is less clear how these signals are made available to OL lineage cells when needed, and how multiple signals are integrated to generate the correct amount of myelin that is needed in a given neural network state. Here we review the pleiotropic effects of some of the extracellular signals that affect myelination and discuss the cellular processes used by the source cells that contribute to the variation in the temporal and spatial availability of the signals, and how the recipient OL lineage cells might integrate the multiple signals presented to them in a manner dialed to the strength of the input.

## Introduction

**Figure 1:**
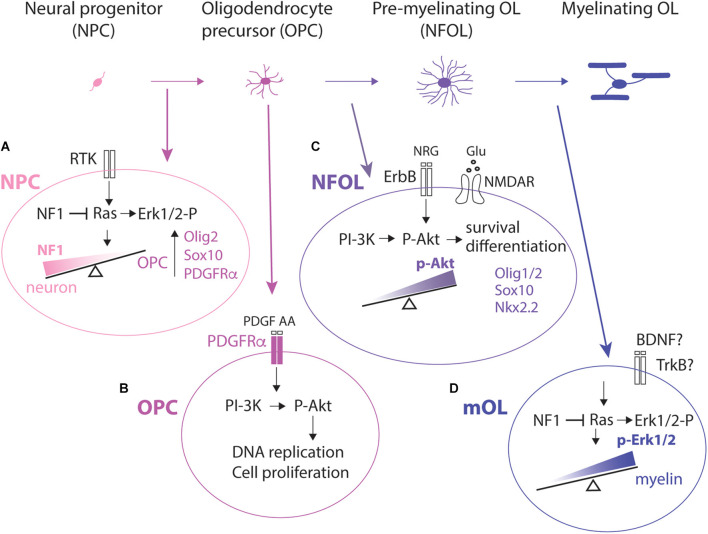
Lineage progression from an NPC to a myelinating OL and examples of key signaling pathways at each stage. **(A)**: NPC. OPCs are specified from NPCs in the ventricular and subventricular zones. Reduction in NF1 leading to enhanced activation of Ras-Mapk-Erk1/2 pathway shifts the fate of NPCs to an OPC fate and suppresses the neuronal fate. Committed OPCs upregulate Olig2 and Sox10 which in turn turns on Pdgfra transcription. **(B)**: OPC. OPC proliferation is dependent on PDGFRα and is mediated by PI-3K activating Akt and not through Mapk activating Erk1/2. **(C)**: NFOL. In NFOLs, PI-3K activating Akt is primarily responsible for their terminal differentiation, survival, and initiation of myelination, possibly *via* the transcription factor FoxO1. HDAC1/2 activity, Myrf, and upregulation of Sox10, and Nkx2.2 are also critical for terminal differentiation. **(D)**: mOL. In myelinating OLs (mOLs), activation of the Ras-Erk1/2 pathway is critical for maintaining myelination. NF1, neurofibromin; RTK, receptor tyrosine kinase; Erk1/2-P, phosphorylated Erk1/2; PDGF, platelet-derived growth factor; PDGFRα, platelet-derived growth factor receptor alpha; PI-3K, phosphatidyl inositol-3-kinase; P-Akt, phospho-Akt; BDNF, brain-derived neurotrophic factor; NFOL, newly formed OLs; HDAC1/2, histone deacetylase 1 and 2; Myrf, myelin regulatory factor; NRG, neuregulin.

In the central nervous system (CNS) oligodendrocyte precursor cells (OPCs) are the lineage committed precursor cells that generate myelinating oligodendrocytes (OLs) and enable saltatory conduction of action potentials. Recent studies show that myelin is not merely a static stack of OL membranes but continues to undergo dynamic remodeling in the adult CNS (Bonetto et al., 2021; Nishiyama et al., 2021). Consistently, OPCs persist throughout the life of the animal, beyond the stage of developmental myelination. Several key steps must occur in order for myelin to be synthesized (Figure 1). First, OPCs must be generated by OL commitment and differentiation from neural precursor cells (NPCs) that reside in discrete areas of the germinal zones and by self-renewal of existing OPCs in the CNS parenchyma. Subsequently, OPCs must proliferate and migrate to occupy their final destination and undergo terminal differentiation into OLs. Terminal OL differentiation is an asynchronous process. Thus, OPCs that undergo self-renewal co-exist with those that terminally differentiate or OLs that are beginning their myelination process. Furthermore, the temporal dynamics of OL appearance and myelination differ in different tracts (Lynn et al., 2021). Finally, newly generated OLs must find target axons and initiate the process of myelination.

Each of these steps is subject to regulation by intrinsic and extrinsic factors. A large body of literature exists on the role of individual factors and the stage at which they regulate OL development and myelination. However, it is less clear how the relevant set of extrinsic factors are presented to OL lineage cells at the correct time and location, and how a response is elicited only in a subset of OL lineage cells and not in others nearby. Moreover, the same single signaling molecule is often used to initiate different cellular programs. In this review, we will first describe the key stages in OL lineage progression that are influenced by the external environment and identify areas where we have a limited understanding of the sources of these external signals and how OL lineage cells integrate them to trigger a specific program. Following a short review of how macromolecules, particularly proteins, are trafficked and released externally, we provide examples of the pleiotropic effects of some proteins that affect OL lineage cells and ways in which their availability can be modulated.

## Dynamic Regulation from NPCs to Myelination

### From NPCs to OPCs

NPCs exist in different germinal zones throughout the neuraxis, where they are specified by different transcription factors in a region- and age-specific manner (Woodruff et al., 2001). In the spinal cord, OLs are initially specified in the pMN and P3 domains of the ventral ventricular zone defined by transcription factors Olig1, Olig2, and Nkx2.2 (Sun et al., 2001; Fu et al., 2002). In the forebrain, early OL specification occurs ventrally in the medial and lateral ganglionic eminences defined by the homeodomain transcription factors Nkx2.1 and Gsx2, respectively (Kessaris et al., 2006). Olig1 and Olig2 are induced in these domains, and this lineage is shared with interneurons (Petryniak et al., 2007). Subsequently, OPCs are generated from the dorsal germinal zones in both the spinal cord and telencephalon. The earlier ventrally generated OL lineage cells migrate dorsally and become intermingled with dorsally generated cells.

In the forebrain, the dorsal wall of the lateral ventricles continues to produce OPCs in the corpus callosum in the adult (Ortega et al., 2013). Newly produced OPCs gradually migrate dorsally and lose the stem cell/pluripotent transcription factor Sox2 as they mature (Kondo and Raff, 2004; Dai et al., 2015), although the role of Sox2 in the oligodendrocyte lineage is age- and context-dependent (Zhao et al., 2015; Zhang et al., 2018). How external signals within the SVZ niche induce OL commitment in a subset of NPCs remains poorly understood. Dynamic oscillation in the intracellular concentration of basic helix-loop-helix transcription factors such as Olig2 (Imayoshi et al., 2013) would provide an explanation of the seemingly stochastic nature in which OPCs are committed from NPCs. Neurofibromin, whose mutation leads to Neurofibromatosis type 1, is encoded by the *NF1* gene and is a Ras-GTPase-activating protein. It negatively regulates OPC proliferation (Bennett et al., 2003) and suppresses OL specification from NPCs in the SVZ (Wang et al., 2012; Figure 1A, NPC). Biallelic deletion of *Nf1* in NPCs switches the fate of NPCs from neurons to OPCs, leading to an increased density of OLs in the developing and adult SVZ. This can be reversed by inhibiting the extracellular signal-regulated kinase Erk1/2, which is a downstream effector of Ras (Wang et al., 2012). It remains unclear what the specific extracellular signal is that directs NPCs to follow an OPC fate and how the signal is transduced in NPCs to activate the OPC program. Paradoxically, Erk1/2 signaling in differentiated OLs is essential for myelination (Ishii et al., 2019) but is dispensable for OPC proliferation (Hill et al., 2013).

### OPC expansion and “maturation”

Once NPCs become committed to the OL lineage, they begin the transcriptional program of pre-OPCs and weakly upregulate platelet-derived growth factor receptor alpha (PDGFRα; Marques et al., 2018; Weng et al., 2019). They subsequently become more permanently committed to OPCs by robustly upregulating the signature OPC transcripts *Pdgfra* and *Cspg4* encoding NG2. As OPCs mature and migrate away from the germinal zone, they lose the expression of NPC molecules such as Sox2 and become fully committed to the OL lineage. This process occurs gradually as they migrate through the parenchyma with a lag period following the initial OL commitment in the SVZ.

Developmental OPCs proliferation is dependent on platelet-derived growth factor AA (PDGF AA), which is a critical mitogen for OPCs and activates PDGFRα on OPCs (van Heyningen et al., 2001; Hill et al., 2013). There are mechanisms that modulate the mitogenic effects of PDGF AA. For example, entrapment of PDGF AA in the extracellular matrix could alter their availability to cells (Pollock and Richardson, 1992) and its co-receptors, such as neuropilin-1 expressed by activated/amoeboid microglia, can trans-activate PDGFRα on neighboring OPCs (Sherafat et al., 2021). In organotypic slice cultures, parenchymal OPCs no longer proliferate in response to other mitogens such as fibroblast growth factor-2 (FGF2) or epidermal growth factor (EGF; Hill et al., 2013). While OPC production in the embryonic spinal cord is dependent on Erk1/2 signal (Newbern et al., 2011), PDGF-dependent OPC proliferation in slice cultures and OPC proliferation *in vivo* are mediated by Akt activation and not by Erk1/2 signal (Ishii et al., 2012; Hill et al., 2013; Figure 1B, OPC). Erk1/2 appears to play a more prominent role in OPC proliferation in dissociated cultures (McKinnon et al., 1990), suggesting the importance of the pericellular microenvironment in regulating signal transduction from PDGFRα in OPCs.

OPCs remain proliferative throughout life. However, as the animal matures, OPCs become more “OL-like” by acquiring a low level of expression of OL-specific genes (Moyon et al., 2015; Marques et al., 2018). These OL-like OPCs are more prevalent in the spinal cord than in the brain (Marques et al., 2018). Besides the signature proteins PDGFRα and NG2 on their surface, OPCs also express voltage-dependent ion channels and neurotransmitter receptors, including AMPA and GABA receptors (Larson et al., 2016), which are essential for their depolarizations at neuron-OPC synapses. There have been a number of studies on whether and how activation of ionotropic glutamate and GABA receptors on OPCs affects their proliferation or OL differentiation (Gallo et al., 1996; Kougioumtzidou et al., 2017; Chen et al., 2018), but the findings vary, and we do not yet fully understand how the signal is transduced following synaptic stimulation of OPCs. While cumulative labeling of cycling cells with thymidine analogs indicates that almost all OPCs are cycling (Psachoulia et al., 2009; Kang et al., 2010; Young et al., 2013), single cell RNA-seq data indicate that cycling OPCs represent only a subset of OPCs (less than 25% of the cells in the total OPC cluster; Marques et al., 2018). *In vivo* multi-color cell fate mapping has revealed that some isolated clones of OPCs undergo a burst of expansion later in adulthood (Garcia-Marquez et al., 2014), suggesting considerable heterogeneity in the mitotic activity of individual OPCs. It remains unknown whether the mitotic competence of individual OPCs is determined intrinsically, for example by the number of cell divisions, or whether it is influenced by the microenvironment surrounding OPCs in specific regions.

### Terminal OL differentiation

The mechanisms that dictate whether individual OPCs differentiate into OLs or continue to self-renew are not fully understood. The signal must be local to their pericellular microenvironment, as OPCs that generate OLs are intermixed with OPCs that self-renew. However, there are some differences in different neuroanatomical regions. In regions where large numbers of myelinating OLs must be generated over a relatively short period of time, such as the white matter in early postnatal CNS, OPCs are more likely to differentiate symmetrically into two OLs, whereas those in gray matter and in older animals are more likely to undergo self-renewing divisions to produce one OL and one OPC or two OPC progeny (Zhu et al., 2011). Many extrinsic signals regulate OL differentiation when tested individually *in vitro* or *in vivo* (Emery, 2010; Huang et al., 2013). However, it remains a major challenge to understand which of the individually tested signals are available to differentiation-competent OPCs at the right time and right place, and what enables them to trigger a response in OPCs that activates specific intracellular signal transducers, transcription factors, and chromatin/DNA regulators to elicit specific outcomes (Huang et al., 2013).

Upon terminal differentiation, OPCs permanently exit the cell cycle, lose PDGFRα and NG2 from the cell surface, and begin the program to terminally differentiate into postmitotic OLs. This process is initiated by the downregulation of PDGFRα and subsequent upregulation of *Nkx2.2* (Zhu et al., 2014). These events, along with upregulation of the myelin regulatory factor (*Myrf*) and transcription factor *Sox10* as well as the function of histone deacetylases HDAC1 and 2, are required for OL differentiation and subsequent upregulation of myelin genes (Emery and Lu, 2015; Liu et al., 2016; Sock and Wegner, 2021). Specific miRNAs downregulate PDGFRα (Budde et al., 2010; Dugas et al., 2010; Zhao et al., 2010), but PDGFRα downregulation and cessation of OPC proliferation are not necessarily coupled with OL differentiation. For example, guanidine compounds that repress *Pdgfra* transcription and OPC proliferation do not stimulate OL differentiation (Medved et al., 2021). Activation of Akt (Protein kinase B) and mammalian target of rapamycin (mTOR) is critical for OL differentiation (Bercury et al., 2014; Ishii et al., 2019), and the effect is mediated by FoxO1 (Wang et al., 2021; Figure 1C, NFOL).

### From newly formed OLs (NFOLs) to myelinating OLs

#### Pre-myelinating OLs

NFOLs have a transcriptomic signature that distinguishes them from mature myelinating OLs (Marques et al., 2016; Tasic et al., 2016). Morphologically they have numerous fine radially oriented processes that appear different from the smaller number of thicker, straight myelinating processes characteristic of mature myelinating OLs. These postmitotic NFOLs that are not yet myelinating OLs are often referred to as premyelinating OLs (Trapp et al., 1997; Hughes and Stockton, 2021) or “lacy OLs” (Zerlin et al., 2004). During the premyelinating stage, NFOLs actively make contacts with target axons before they begin the program of myelination (Hughes and Stockton, 2021). They express the DM20 splice variant of the myelin tetraspanin protein proteolipid protein (PLP; Trapp et al., 1997). NFOLs in white matter tracts differentiate quickly into myelinating OLs, whereas those in gray matter stall in the premyelinating stage longer (Zerlin et al., 2004).

NFOLs seem to be particularly sensitive to their local environment for their survival, and as many as 50% of them die (Barres et al., 1992). When OPC production is increased by increasing the amount of available PDGF, supernumerary OLs die (Calver et al., 1998). Conversely, increasing the number of axons in the optic nerve leads to increased OL numbers (Burne et al., 1996), suggesting that axonal signals regulate the survival of NFOLs as a part of a strong homeostatic mechanism that maintains a constant density of OLs optimized to the local neural circuit function.

In the early postnatal forebrain, terminal OL differentiation occurs within a temporal window of 3–4 days after the last OPC division in both gray and white matter, and the fate of NFOLs is influenced by their microenvironment (Hill et al., 2014). For example, reduction of myelin accelerates terminal OL differentiation after the final OPC mitosis, while loss of whisker sensory input reduces the number of NFOLs that survive (Hill et al., 2014). Conversely, in adults, whisker stimulation increases the survival of NFOLs (Hughes et al., 2018). Direct electrical stimulation of cultures containing OPCs and axons promotes the survival of OLs (Gary et al., 2012). Loss of GRIA2 (*GluR2*) and GRIA3 (*GluR3*) AMPA receptor subunits compromises OL survival (Kougioumtzidou et al., 2017). Interestingly, *Nfatc4* (nuclear factor of activated T-cells cytoplasmic 4), which is activated by the Ca^2+^-dependent protein phosphatase calcineurin, de-represses *Olig2* and *Nkx2.2* in a *Sox10*-dependent manner (Weider et al., 2018). Thus, neuron-derived signals could change [Ca^2+^]_i_ and trigger the signals that downregulate *Pdgfra* mRNA and upregulate *Nkx2.2* and subsequent progression toward myelinating OLs. These events likely depend on the pattern of neuronal activity (Nagy et al., 2017), and thus they are likely to differ in different regions (Etxeberria et al., 2016 ) and expression of voltage-dependent Ca^2+^ channels (Cheli et al., 2016; Pitman et al., 2020).

#### Myelination

Interestingly, the process of myelin ensheathment itself requires no extrinsic input from other CNS cell types. *In vitro*, primary OLs have been shown to contact and wrap inert nanofibers mimicking the 3D structure of axons but lacking any signaling cues that would normally be present during myelination (Lee et al., 2012). *In vivo*, however, the process of axonal selection and myelination is still highly regulated, and external factors exert a major influence over both the timing and extent of myelination.

*Nkx2.2* is re-upregulated as myelination proceeds (He et al., 2009). The process of myelin ensheathment requires active axons undergoing regulated exocytosis (Hines et al., 2015; Mensch et al., 2015). While PI3K-Akt signal through mTORC1 is critical for OL differentiation and the initiation of myelination, Erk1/2 alone plays a critical role in maintaining myelin. For the process of generating myelin sheath, both Akt and Erk1/2 pathways are needed to effect mTORC1 (Ishii et al., 2019; Figure 1D, mOL).

In addition to playing a major role in regulating the survival of NFOLs, neuronal activity has also been shown to influence myelination and OL maturation. Pharmacological stimulation of individual neurons increases the probability of their axons being myelinated (Mitew et al., 2018), and repeated photostimulation, but not a single 3-h stimulation, of cortical association neurons, promotes remyelination of callosal axons and improves conduction (Ortiz et al., 2019). Single photostimulation, which robustly induces OPC proliferation (Gibson et al., 2014), may result in a moderate increase in OLs but may not be sufficient to allow progression to trigger the myelination program. Enhanced sensory experience has also been shown to promote OL maturation and differentiation (Forbes et al., 2020; Goldstein et al., 2021). How changes in neuronal activity are detected by NFOLs remains to be solved. Synapses that are present in OPCs are quickly disassembled at the onset of differentiation (De Biase et al., 2010; Kukley et al., 2010). However, the vesicular release of ATP and glutamate from axons has been shown to evoke Ca^2+^ transients in OPCs (Ziskin et al., 2007; Hamilton et al., 2010), and both have been suggested to play a role in regulating OL differentiation and myelination, suggesting that NFOLs may also utilize extra-synaptic mechanisms to sense neuronal activity (Agresti et al., 2005; De Biase et al., 2010; Kukley et al., 2010; Wake et al., 2011, 2015).

## Trafficking of Cell Surface and Secreted Molecules

**Figure 2:**
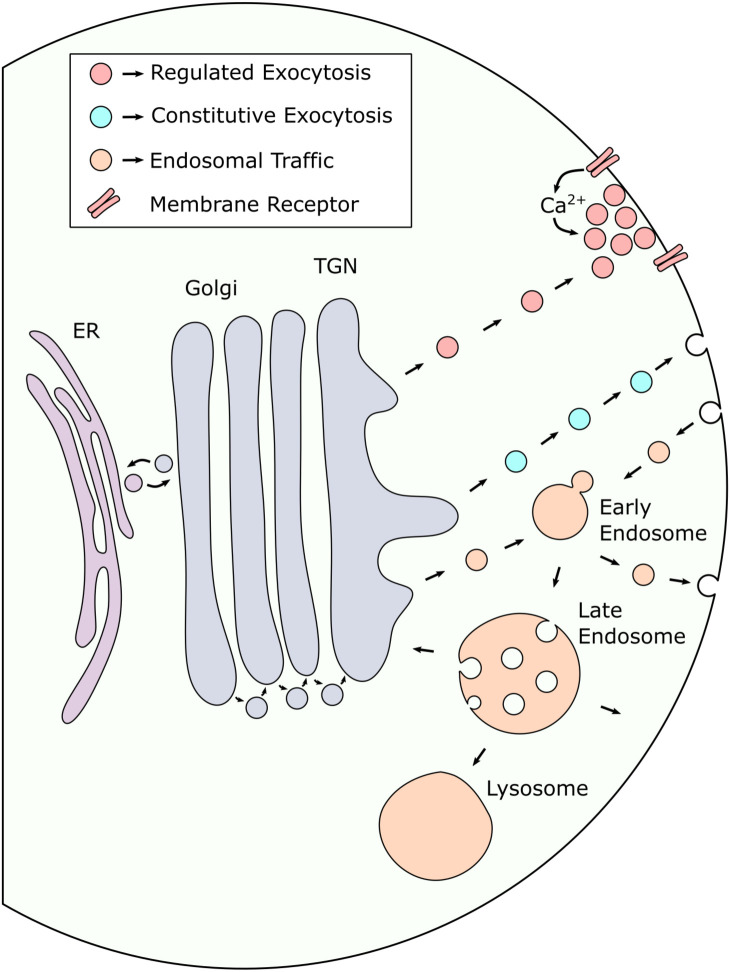
Secreted proteins are trafficked to the cell surface *via* multiple distinct pathways. After synthesis in the endoplasmic reticulum (ER), proteins destined for exocytosis undergo processing in the Golgi before being sorted into intracellular transport vesicles in the trans-Golgi network (TGN). Transport vesicles leaving the TGN can either be targeted directly to the plasma membrane to undergo regulated or constitutive exocytosis or be transported to the endosomal pathway, either for further sorting prior to exocytosis or to deliver endosome specific cargo. Proteins that are retrieved from the cell surface are targeted to early endosomes (EE) which act as sorting platforms in endosomal traffic to and from the membrane. EEs can mature into late endosomes (LE) which act as a final sorting station for proteins destined for lysosomal degradation. LEs can also mature into multi-vesicular bodies (MVB) which can alternatively fuse with the plasma membrane to facilitate exosome release. Small arrowheads indicate the direction of vesicle traffic.

OL lineage cells at different stages of development integrate and respond to the variety of autocrine and paracrine signals presented to them in distinct ways. Their ability to respond, and the nature of their response, are largely determined by the complement of cell surface proteins and secreted signaling molecules present in each stage. Moreover, some signals are tonically or diffusely present in their microenvironment, while others are only made available in a spatially restricted manner. Thus, precisely regulated trafficking and secretion of key signaling factors are essential for proper OL lineage development. Figure 2 summarizes the major components of the secretory pathways involved in trafficking protein cargo to the cell surface.

In most cases, proteins that are destined for the cell surface, either to be incorporated into the membrane or released into the extracellular space, are first synthesized in the endoplasmic reticulum before being transported to the Golgi apparatus. Following passage through the Golgi, vesicles carrying protein cargo are trafficked through the trans-Golgi network (TGN) where they are sorted into transport vesicles targeting them to appropriate subcellular destinations. After leaving the TGN, vesicles carrying protein cargo can either be transported directly to the plasma membrane for exocytosis or be targeted to the endosomal pathway for further sorting.

Exocytosis can occur either constitutively, where vesicles carrying protein cargo fuse immediately upon arriving at the plasma membrane, or in a regulated fashion, where transport vesicles are stored and only released in response to a defined stimulus (Stöckli et al., 2016). During regulated secretion, the stimulus involved often results in a rise in intracellular Ca^2+^, which acts as a second messenger to trigger the fusion of primed secretory vesicles with the plasma membrane. Distinct pools of these vesicles have been observed with differing degrees of Ca^2+^ sensitivity, depending on their complement of associated regulatory proteins. Although constitutive exocytosis requires no stimulus to occur, the rate of constitutive secretory processes can still be regulated at the cellular level *via* changes in gene or protein expression. This occurs in B lymphocytes, which are stimulated to produce large amounts of antibody which is then secreted constitutively (Borghesi and Milcarek, 2006).

Proteins that exert specific effects on OL lineage cells can be exocytosed from the cell of origin by a variety of mechanisms. Where they are ultimately targeted determines the mode of activation. The availability of secreted proteins is dependent on whether they are freely available to bind to receptors on OPCs, need a co-receptor to activate the receptor on OPCs, or whether they become sequestered in the extracellular matrix and require an additional mechanism to encounter the receptor on OPCs. The duration of the effect is determined by the half-life of the protein outside the cell and the rate of reuptake, if it occurs. In the case of cell surface proteins, the duration of the availability is additionally influenced by the rate of their endocytosis. For example, PDGFRα is rapidly internalized in the presence of the ligand in 5–30 min, whereas its half-life is longer than 2 h in resting cells (Kazlauskas, 2017).

### SNARE proteins in exocytosis

Soluble N-ethylmaleimide-Sensitive Factor Attachment Protein (Fretto et al., 1993) receptors (SNAREs) are a highly conserved family of small membrane-anchored proteins which comprise the minimal molecular machinery required for membrane fusion involved in exocytosis and endosomal trafficking (Jahn and Scheller, 2006; Galli et al., 2013). The mechanism of SNARE-mediated exocytosis has been most extensively studied in the context of regulated exocytosis at the neuronal presynapse, where the local influx of Ca^2+^ can trigger vesicle fusion on the order of 1 ms. The discovery of a core SNARE complex in neurons, consisting of Syntaxin 1 (STX1), SNAP-25, and vesicle-associated membrane protein 2 (VAMP2), ultimately led to the development of the SNARE hypothesis, which proposed that membrane fusion is mediated by the binding of vesicle-associated SNAREs (v-SNAREs) to cognate target membrane-associated SNAREs (t-SNAREs).

SNARE proteins are required for both regulated and constitutive exocytosis, and although SNARE proteins themselves are not the primary drivers of vesicle targeting, specific combinations of SNARE proteins in a cell type are often linked to specific secretory pathways or processes (Kasai et al., 2012). Therefore, experimental modulation of defined SNARE proteins can serve as a tool to better understand the role of specific secretory processes in a particular cell type. The light chains of different clostridial neurotoxins, which include tetanus toxin and botulinum neurotoxins, cleave distinct sets of SNARE proteins with a high degree of specificity (Schiavo et al., 2000). Cell-type specific expression of these toxins has primarily been employed as a means of disrupting SNARE-mediated exocytosis in different neuron subtypes, though it has also been used to investigate the role of gliotransmission in Müller cells of the retina (Slezak et al., 2012). The time course of SNARE-dependent release in response to a stimulus differs among different cell types, with synaptic vesicles representing the fastest type of release on the order of milliseconds, while release from dense core vesicles at some synapses and neuroendocrine cells are 2– orders of magnitude slower (Kasai et al., 2012). Stimulated SNARE-dependent release from astrocytes is three orders of magnitude slower than that of presynaptic vesicle release from axons (Schwarz et al., 2017). Thus, proteins released from both astrocytes and neurons, such as brain-derived neurotrophic factor (BDNF; Zhang et al., 2014), could be presented to OPCs by different dynamics and could lead to the activation of distinct signaling pathways. Another example is PDGF AA, which is produced by neurons, OLs, and microglia (Zhang et al., 2014), and could lead to differential activation of the downstream intracellular signaling pathway from PDGFRα on OPCs depending on the cellular source, mechanism of release, and the quantity of release from the different cells surrounding OPCs.

### SNARE-mediated exocytosis in OL lineage cells

OL lineage cells employ SNARE-mediated membrane fusion to enable secretion, vesicle, and membrane trafficking, as well as targeting and sorting of membrane proteins, which provide crucial functions at each of the different stages in OL development (Feldmann et al., 2011). Recently, we and others have shown that genetic expression of Vamp1, 2, and 3-specific B type botulinum toxin (BoNT/B), in OPCs using *Cspg4-cre* (Fekete et al., 2022) or *Pdgfra-creERT* (Pan et al., 2022) or in newly differentiated OLs using *Cnp-cre* (Lam et al., 2022) leads to severe hypomyelination due to inability of the Vamp2/3-cleaved OLs to produce myelin membranes. These findings suggest that a critical autocrine factor that is either secreted from newly generated OLs or is inserted into the plasma membrane of new OLs plays a critical role in myelination.

SNARE-mediated processes in the OL lineage have also been shown to regulate the transcription of myelin basic protein (MBP), a peripheral membrane protein localized at the cytoplasmic surface that is critical for myelin compaction (Bijlard et al., 2015). *Mbp* mRNA is translated locally at the plasma membrane after being transported from the soma to the myelin membranes in RNA granules (Ainger et al., 1997; Barbarese et al., 1999). When the t-SNARE gene *Stx4* is knocked down or overexpressed, *Mbp* transcription is severely reduced, with no apparent effects on OL viability or the expression and trafficking of the other myelin proteins PLP and CNP (2’,3’-cyclic nucleotide phosphodiesterase; Bijlard et al., 2015). Knocking down *Stx4* in mature OLs after MBP is translated has no effect on the level of MBP in the myelin. Knockdown of *Vamp3* encoding the v-SNARE partner for STX4 does not alter the trafficking of *Mbp* mRNA. Intriguingly, the conditioned medium of normal developing OLs restores *Mbp* transcription in *Stx4*-downregulated cells, suggesting that STX4 is critical for the release of an autocrine factor that is necessary for initiating *Mbp* transcription.

Myelin proteolipid protein (PLP), a major tetraspanin membrane protein in myelin, is synthesized in the endoplasmic reticulum and is trafficked *via* two distinct SNARE-dependent processes: (1) mediated by VAMP3 and its cognate t-SNARE partner STX4 *via* recycling endosomes; and (2) mediated by VAMP7 and its partner STX3 *via* late endosomes and lysosomal compartments (Feldmann et al., 2011). Compromised VAMP3 or VAMP7 function in cultured OLs leads to loss of PLP from the cell surface and its accumulation in the soma, but these manipulations do not affect the total expression level of PLP. During OL maturation, endocytic turnover of PLP decreases as the level of PLP at the myelin membrane surface increases, and this is accelerated in the presence of neurons *via* cAMP (Trajkovic et al., 2006).

What triggers SNARE-mediated exocytosis in OL lineage cells remains unknown. When OPCs receive synaptic inputs from neurons, they respond to vesicularly released glutamate through their AMPA receptors, which consist of Ca^2+ ^-permeable subunits (Bergles et al., 2000; Ge et al., 2006). Voltage-gated Ca^2+^ channels and Na^+^ /Ca^2+^ -exchanger also contribute to an increase in [Ca^2+^]_i_ in OPCs (Tong et al., 2009; Cheli et al., 2016). While an increase in [Ca^2+^]_i_ can be readily detected after direct stimulation of AMPA receptors on OPCs, or by activating DRG neurons in a DRG-OPC coculture (Wake et al., 2011), demonstration of a local rise in [Ca^2+^]_i_ in response to physiological synaptic stimulation of OPCs has not been achieved (Haberlandt et al., 2011; Sun et al., 2016). Regardless of the nature of the extracellular stimulus that elicits a rise in [Ca^2+^]_i_ in OPCs, the importance of Ca^2+^ signal in OPCs is evident. For example, the deletion of voltage-dependent Ca^2+^ channels Cav_1.2_ and Cav_1.3_ in OPCs reduces OL differentiation and myelination in white matter (Cheli et al., 2016) and reduces OPC proliferation in the cortex (Zhao et al., 2021). Furthermore, Nfat proteins, which are activated by the Ca^2+^-dependent phosphatase calcineurin, cooperate with Sox10 to activate Nkx2.2, which is critical for OL differentiation (Weider et al., 2018). One could thus hypothesize whether depolarization and Ca^2+^ entry into OPCs at neuron-OPC synapses could trigger specific SNARE proteins in OPCs and trigger them to release molecules that are critical for OL lineage progression and possibly those that in turn affect neurons in a paracrine fashion.

## Dynamic Modulation of Extracellular Signal Availability

Many signaling molecules exert a wide range of effects depending on the context in which they are presented to the target cell. Their effects depend on their temporal and spatial availability and the distribution of their receptors. In this section, we will use brain-derived neurotrophic factor (BDNF) and L1CAM (L1 cell adhesion molecule) as examples to illustrate the different ways in which soluble and integral membrane proteins might be delivered to OL lineage cells. We chose to discuss these proteins in detail because their expression and availability are regulated in different ways, including neuronal activity, and they exhibit pleiotropic cellular effects. While their functions have been described extensively for neurons, some of these regulatory mechanisms may also be applied to how they regulate OL differentiation and myelination. We also discuss the role of the intracellular Src family kinase Fyn as an integrator of multiple signals to OL lineage cells and its potential role as a filter for eliciting specific responses in newly differentiated OLs.

### Pleiotropic effects of the soluble protein BDNF

#### Diverse effects of BDNF on neurons

**Figure 3:**
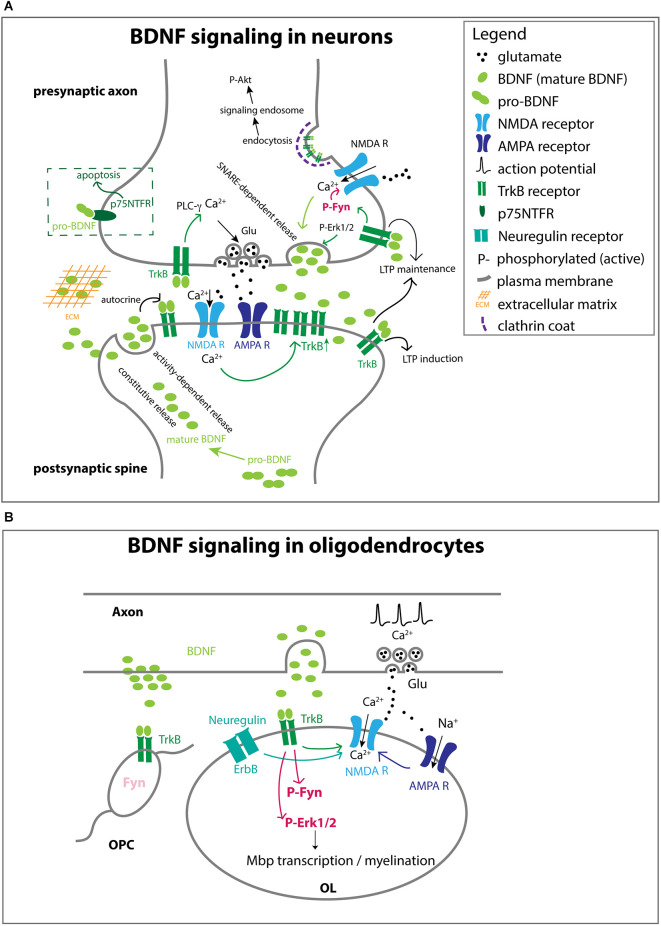
Different ways in which BDNF acts on cells **(A)**. BDNF-TrkB function at the neuronal synapse. BDNF from the target neuron or other cells in the target area mediates the survival and axon growth of the presynaptic neuron. Some of the effect is local, while other effects may be mediated by clathrin-dependent endocytosis of BDNF-TrkB complex that could be transported retrogradely by signaling endosomes, analogous to what has been reported for NGF and its high-affinity receptor TrkA. Mature BDNF can be secreted constitutively or in a regulated manner *via* SNARE-dependent mechanisms. BDNF may not be immediately available to the receptor if it becomes embedded in the extracellular matrix. While the majority of the effects are mediated by mature BDNF, pro-BDNF can also be secreted and act on p75NTFR to elicit a cell death response. BDNF acting on presynaptic and postsynaptic membranes can promote LTP. At the presynaptic terminal, axon depolarizations increase Ca^2+^ entry through NMDA receptors, which in turn activates the SNARE-dependent release of BDNF. The neuronal activity also increases the level of TrkB at the presynaptic surface, and TrkB activation increases Ca^2+^ *via* PLC-γ activation, leading to enhanced glutamate release from presynaptic vesicles. At the postsynaptic membrane, BDNF acting on TrkB also contributes to LTP by increasing NMDA current. BDNF is secreted in an activity-dependent manner and acts in an autocrine fashion on TrkB on the same spine, which is necessary for LTP. Some of the processes indicated in one compartment also apply to the other compartment. The concept of signaling endosomes has been used to explain the function of endocytosed TrkB-BDNF complex that is transported from the internalized site on the axon back to the soma where it exerts developmental effects (Grimes et al., 1996), similar to what had been reported earlier for TrkA-NGF (Grimes et al., 1996). **(B)** BDNF-TrkB function at axonand OL interface. Neuronal activity triggers BDNF release and glutamate release. BDNF binds to TrkB on OLs and activates Fyn and Erk1/2, which promotes the transcription of myelin genes and myelination. NRG acting on the ErbB receptor and BDNF activating TrkB both increase Ca^2+^ permeation through NMDA receptors. Fyn is much less abundant in OPCs than in OLs, even though OPCs express TrkB.

BDNF belongs to the family of neurotrophins. Nerve growth factor (NGF) was the first member of the family, which was discovered as a target-derived trophic factor for sympathetic and sensory neurons in the periphery (reviewed in Levi-Montalcini, 1987). Subsequently, BDNF was isolated and characterized from the pig brain in search for a neurotrophic factor that functions in the CNS (Barde et al., 1982; Leibrock et al., 1989). In most cases BDNF is proteolytically cleaved from pro-BDNF inside the cell to generate mature BDNF, which is secreted (Figure 3A). The binding of mature BDNF to the high-affinity tropomyosin receptor kinase B (TrkB) receptor activates phospholipase C gamma (PLC-γ), PI-3K, and Erk1/2, and exerts a wide range of effects on target neurons such as survival, axon growth, synaptogenesis, and synaptic plasticity (Thoenen, 1995). Under some conditions, pro-BDNF can also be released from the cell. Secreted pro-BDNF binds preferentially to p75 low-affinity neurotrophin receptor (p75^NTFR^) and elicits effects such as apoptosis and long-term depression, which are distinct from the effects of TrkB activation by mature BDNF (Chao, 2003). The cellular effects of BDNF depend on the duration of its availability, the distribution of TrkB receptors, and the subcellular region of the target cell to which BDNF is made available.

The temporal and spatial availability of BDNF is influenced by several factors (Figure 3A). For example, constitutive release provides a tonic level of extracellular BDNF, whereas release regulated by neuronal activity leads to a pulsatile elevation in local BDNF concentration (Balkowiec and Katz, 2002). In the developing callosal axons, BDNF secretion is mediated by the vesicular and membrane SNAREs VAMP2 and SNAP25/47, respectively, and the function of these SNARE proteins is critical for BDNF-mediated axonal differentiation (Shimojo et al., 2015). Once secreted, BDNF has a limited ability to diffuse, since it has a tendency to become embedded in the extracellular matrix because of its positive charge (Park and Poo, 2013). In neuronal culture, different effects of BDNF on spine development are elicited by different temporal patterns of elevated local BDNF concentrations (Wang et al., 2022).

At the synapse, BDNF modulates synaptic plasticity *via* specific pre- and postsynaptic mechanisms (Figure 3A). TrkB receptors at pre- and postsynaptic membranes differentially contribute to long-term potentiation (LTP) in hippocampal neurons (Lin et al., 2021). BDNF binding to TrkB increases [Ca^2+^]_i_ by activating PLC-γ. Activated TrkB also activates Fyn, which in turn increases the open probability of NMDA receptors, additionally contributing to Ca^2+^ influx (Xu et al., 2006; Bjarnadottir et al., 2007; Hildebrand et al., 2016). Increased presynaptic NMDA receptor activity in turn increases [Ca^2+^]_i_, which promotes BDNF release (Lituma et al., 2021). At postsynaptic spines, increased activity increases the release of BDNF, which activates TrkB on the same spine in an autocrine manner and promotes LTP (Wang et al., 2022). Furthermore, neuronal activity increases the number of TrkB receptors on the postsynaptic membrane. BDNF, like NGF, is internalized by clathrin-mediated endocytosis. Signaling endosomes containing endocytosed TrkB-BDNF complex are transported from the internalized site on the axon back to the soma where they promote survival *via* Akt (Grimes et al., 1996).

#### Effects of BDNF on OL lineage cells

BDNF exerts different effects on OPCs and OLs, with a more prominent effect at later stages during myelination. *Bdnf−/−* mice survive up to 4 weeks and show severe hypomyelination with reduced *Mbp* and *Plp1* mRNA in the developing optic nerves (Korte et al., 1995; Cellerino et al., 1997). Heterozygous *Bdnf+/−* mice also exhibit reduced myelin and a transient reduction in OL lineage cell density in the optic nerves (Vondran et al., 2010; Nicholson et al., 2018). Exogenously applied BDNF has no effect on OPC proliferation or OL survival (Barres et al., 1993), although there seems to be some regional heterogeneity in their response to BDNF (Du et al., 2003). However, knockdown of TrkB in purified OPC cultures increases OPC proliferation (Wong et al., 2013), suggesting a suppressive role for TrkB in OPC proliferation. Paradoxically, *Bdnf+/−* mice exhibit impaired OPC proliferation after cuprizone-induced demyelination (Tsiperson et al., 2015; Huang et al., 2020), which could be secondary compensatory proliferation of TrkB-expressing OPCs that occurs in response to myelin defects (Wong et al., 2013). BDNF also increases OPC process extension, especially in the presence of inhibitory proteoglycans (Siebert and Osterhout, 2021). Overall, these effects of BDNF on OPCs are limited and appear only in culture or are reversed as development proceeds.

BDNF exerts a more prominent and long-lasting pro-myelinating effect in OLs *via* TrkB (Barres et al., 1993; Xiao et al., 2010). Conditional knockout of TrkB in postmitotic OLs reduces myelin thickness without affecting OL production or axonal contact (Wong et al., 2013). As described above in section “From newly formed OLs (NFOLs) to myelinating OLs”, myelination occurs in two phases. The first phase does not require neuronal activity. Neuregulin (NRG) increases NMDA receptor currents in OLs, thereby switching to an activity-dependent mode of myelination (Lundgaard et al., 2013). BDNF has a similar effect of enabling activity-dependent myelination by increasing NMDAR currents in OLs. BDNF stimulation of OL TrkB leads to Erk1/2 phosphorylation, and manipulating the strength of Erk1/2 activity directly affects the level of myelin protein expression and myelination *in vitro* (Xiao et al., 2012). Consistently, Erk1/2 knockout reduces developmental myelination (Ishii et al., 2019).

#### Fyn kinase as a transducer of BDNF signal

**Figure 4:**
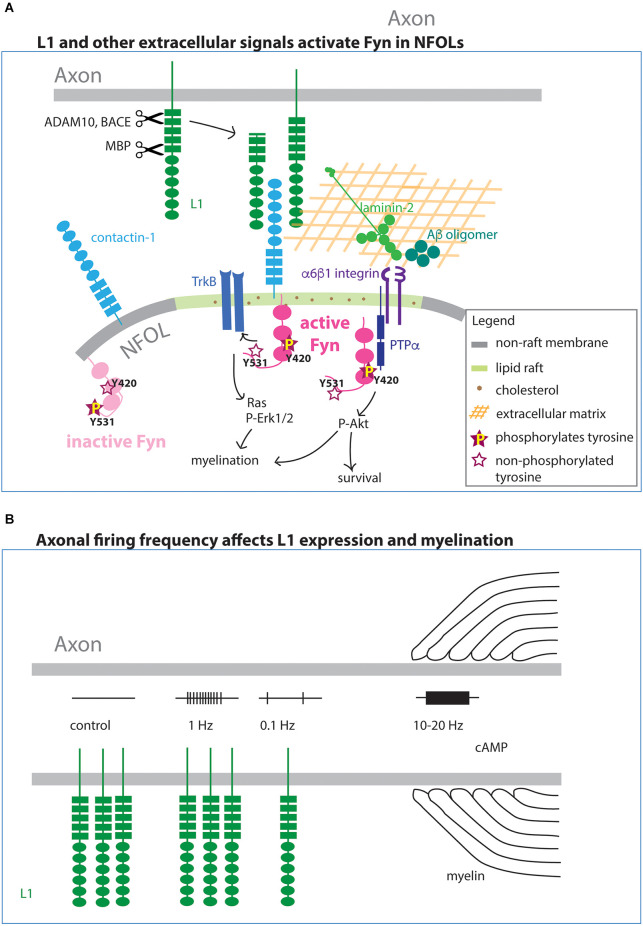
Integral membrane and extracellular matrix affecting myelination *via* Fyn **(A)**. Axonal L1 forms a complex with contactin-1 on OLs. The complex becomes preferentially localized to lipid rafts, where they complex with and activate Fyn by phosphorylated Y420. Fyn in the non-raft membrane is not active (not phosphorylated on Y420 but phosphorylated on the regulatory Y531 site). In addition to the membrane-bound form, cleaved L1 also interacts with contactin-1. In addition to L1, the extracellular molecules laminin-2 and Aβ oligomer also activate Fyn on OLs. Activated Fyn also promotes TrkB function. **(B)** The level of L1 expression on the surface of the axon is regulated by the frequency of action potential firing. High-frequency firing increases myelination, and this effect in some cases is mediated by cAMP.

According to RNA-sequencing datasets, TrkB mRNA is more abundant in OPCs than in OLs (Zhang et al., 2014; Marques et al., 2018). Why then is the effect of BDNF more prominent during myelination and not at the earlier OPC stage? One could speculate that OLs but not OPCs have the critical intracellular mechanism that endows them with a high sensitivity of detecting BDNF to elicit a sustained Erk1/2 response necessary to promote myelination. One candidate for such a stage-specific transducer BDNF/TrkB signal is Fyn kinase (Figures 3B, 4). Fyn is a member of the Src family non-receptor tyrosine kinases. Tyrosine phosphorylation at Y420 increases its kinase activity, whereas tyrosine phosphorylation on Y531 in the C-terminal regulatory region inhibits kinase activity (Matrone et al., 2020). Fyn is marginally expressed in OPCs but robustly upregulated in NFOLs (Colognato et al., 2004; Zhang et al., 2014; Marques et al., 2018). It is sustained at a high level during active developmental myelination (Krämer et al., 1999) and is downregulated by the end of the first postnatal month after the peak of myelination. *Fyn−/−* mice exhibit hypomyelination apparent at 4 weeks (Umemori et al., 1994). In zebrafish, the number of myelin sheaths made by each OL is dependent on Fyn kinase activity (Czopka et al., 2013). In OLs, the ability of BDNF to phosphorylate and activate Erk1/2 is mediated by Fyn (Peckham et al., 2016). Thus, the differential response of OPCs and OLs to BDNF could in part be attributed to the greater expression and activity of Fyn. Since OPCs and OLs coexist in the white and gray matter, the specific effects of BDNF must be determined by how BDNF is made available specifically to individual OPCs or OLs and the temporal and spatial pattern of TrkB expression and downstream effectors in OL lineage cells at different stages. Similar fine-tuning mechanisms are likely to shape the response of OL lineage cells to a variety of other soluble factors such as PDGF, FGF, and Wnt (Emery, 2010; Adams et al., 2021).

### Integral membrane and extracellular matrix proteins that promote myelination *via* Fyn

The importance of axonal activity in myelination has been a subject of intense investigation. Early studies have shown that dark-rearing reduces the number of myelinated axons in the optic nerve (Gyllensten and Malmfors, 1963), and conversely, myelination is accelerated by artificial eye opening (Tauber et al., 1980). Many other studies have also shown that neuronal activity promotes myelination (Barres and Raff, 1993; Demerens et al., 1996; Stevens et al., 2002), but the molecular and cellular mechanisms by which different patterns of neuronal activity initiates the myelination program in OLs is less clear. In this section, we will use the L1 cell adhesion molecule as an example of an extracellular signal that activates Fyn and highlight the multiple ways in which its presentation to OLs can be modulated.

#### L1 cell adhesion molecule functions *via* Fyn in myelination

One proposed mechanism for axon-OL signaling in regulating myelination is an interplay between cell surface adhesion molecules on axons and the adjacent OL processes. L1CAM belongs to the immunoglobulin superfamily of calcium-independent cell adhesion molecules (IgCAM) and is predominantly expressed on developing axons. L1 contains six Ig-like domains (Ig1–6) followed by five fibronectin type III-like domains (FN1–5) in the extracellular domain and a short cytoplasmic domain at the C-terminus (Figure 4A). It engages in both homophilic and heterophilic interactions in cis and trans and is involved in a variety of developmental processes including neurite growth, cell survival, migration, myelination, and synaptic plasticity (Sytnyk et al., 2017).

Binding partners of L1 on NFOLs include contactin-1 (Cntn1), which is structurally related to L1, and integrins (Laursen et al., 2009). Cntn1 is a glycosylphosphatidyl-inositol (GPI)-anchored protein that consists of six Ig domains and four FN type III-like domains. The binding of L1 to Cntn1 promotes myelination through transient activation of Fyn kinase (Figure 4A), and the interaction occurs specifically in lipid rafts (Krämer et al., 1999; Laursen et al., 2009). Lipid rafts are microdomains in the plasma membrane that are enriched in sphingolipids and cholesterol, as well as GPI-anchored proteins, where integral membrane proteins such as cell adhesion molecules and growth factor receptors form detergent-insoluble glycolipid-enriched complexes and function as a platform for cell signaling (Simons and Ikonen, 1997). Lipid rafts are prominent in NFOLs but are not commonly detected in OPCs (Krämer et al., 1999). When Fyn is complexed with Cntn1 and L1 in lipid rafts, its kinase activity is enhanced (Zisch et al., 1995; Krämer et al., 1999). Conversely, outside lipid rafts, Fyn is not complexed with Cntn1 or L1, and it is phosphorylated on the regulatory tyrosine at Y531, which makes it inactive (Figure 4A). One downstream consequence of Fyn activation is the local translation of *Mbp* mRNA (White et al., 2008; Kramer-Albers and White, 2011), leading to MBP accumulation at the inner plasma membrane leaflet, where it plays a critical role in myelin compaction.

#### Activity-dependent neuronal expression of L1

The expression of L1 is dynamically regulated. In cultures of dorsal root ganglion (DRG) neurons, L1 expression is tightly regulated by the frequency of action potential firing (Figure 4B). L1 expression is significantly reduced when axons are stimulated at 0.1 Hz but remains unaltered when stimulated at 1 Hz (Itoh et al., 1995). Stimulation of neuron-OL cocultures at 10–20 Hz increases the number of MBP+ myelin sheaths and reduces apoptotic OLs, whereas 1 Hz stimulation has no change (Stevens et al., 2002; Gary et al., 2012). Furthermore, the effect of the 10–20 Hz stimulation is mediated by cAMP (Gary et al., 2012; Malone et al., 2013). These observations provide evidence that axons, and consequently OLs, have a molecular mechanism to detect and transduce different levels of neuronal activity. Additional studies are needed to obtain a more complete understanding of intensity-dependent signaling.

#### Proteolytic cleavage of L1

L1 is highly expressed on unmyelinated axons before and at the time of contact with OL processes but is quickly downregulated from the entire axon when one or more axon segments become ensheathed by OLs (Bartsch et al., 1989; Barbin et al., 2004). It is not known how myelination signals to axons to mediate this downregulation. It is possible that the rapid loss of L1 immunoreactivity from the surface of axons is caused by the shedding of L1. Some proteolytically cleaved forms migrate away from the site of cleavage, whereas other cleaved forms are retained at the membrane (Kleene et al., 2021). Notably, soluble L1 interacting with Cntn1 can activate Fyn in OLs (White et al., 2008).

L1 is proteolytically cleaved by a number of different enzymes including ADAM10 and 17 (a distintegrin and metalloproteinase domain-containing protein 10 and 17), BACE1 (β-site amyloid precursor protein-cleaving enzyme), and externally released MBP (Figure 4A; reviewed in Kiefel et al., 2012), eliciting distinct cellular responses. BACE1, which is a type 1 transmembrane aspartic protease, cleaves amyloid precursor protein (APP) to generate Aβ peptide. In mice, *Bace1* transcript is most abundantly detected in NF OLs (Zhang et al., 2014; Marques et al., 2018). When BACE1 is inhibited in primary neuronal cultures, there is a significant reduction of L1 and other cell adhesion molecules in the secretome, including the close homolog of L1 (CHL1), which is expressed on OPCs as well as neurons (Zhou et al., 2012; Zhang et al., 2014). BACE1 interacts with ADAM10, and ADAM10 enhances the catalytical activity of BACE1 to cleave CHL1 (Wang et al., 2018). *Bace1* knockout mice exhibit hypomyelination in the adult hippocampus (Hu et al., 2006) but not in the corpus callosum (Treiber et al., 2012), suggesting a potential role for BACE1 in axon-OL interaction, perhaps through its ability to regulate the amount of L1 available to interact with Cntn1 and stimulate Fyn on OLs. L1 is also cleaved by extracellular MBP. In addition to its well-known intracellular role in myelin compaction, MBP can be released out of the cell as a fusion with the C-terminal region of dynamin 1 or dynamin 1-related protein. Extracellular MBP cleaves L1 at a site in the FN1 domain and affects L1-mediated neurite growth and cell survival (Lutz et al., 2014; Kleene et al., 2021).

Knockdown of the l1cam-b in zebrafish significantly reduces the number of myelinated fibers and oligodendrocytes (Linneberg et al., 2019). In DRG-OL cocultures soluble L1 extracellular domain (ECD) promotes myelination. L1 ECD rescues the myelination defects caused by metalloproteinase inhibitors, suggesting that the shedding of L1 enhances myelination (Linneberg et al., 2019). In addition to the cleaved products, L1 is also found in exosomes, which suggests that it could function at remote sites away from its site of synthesis and surface expression.

#### Other extracellular signals that activate Fyn

Several extracellular matrix proteins are known to activate Fyn in OLs. The amyloid Aβ oligomer promotes OL maturation and transport of *Mbp* mRNA to the distal processes by interacting with integrins and subsequently activating Fyn (Quintela-López et al., 2019). In neurons, Fyn targets TrkB to lipid rafts (Pereira and Chao, 2007). A similar lateral movement within the plasma membrane could contribute to converging the effects of L1, laminin, and BDNF in NFOLs.

Fyn is also involved in transducing the signal presented to NFOLs by laminin-2 (merosin), which is a large extracellular matrix molecule whose reduction causes delayed OL maturation and muscular dystrophy (Relucio et al., 2009). Laminin-2 binds to α6β1 integrin on NFOLs, which interacts with and activates Fyn (Colognato et al., 2004; Relucio et al., 2009). NRG promotes the survival of NFOL, and this effect is amplified when the cell interacts with laminin-2 (Colognato et al., 2004). Engagement with laminin-2 reduces phosphorylation at Y531 in the regulatory subunit of Fyn, leading to increased Fyn kinase activity. Without laminin-2, the survival effect of NRG is mediated by PI-3K, whereas engagement of the cells with laminin-2 switches the signaling to the MAPK-Erk1/2 pathway. The cell surface protein tyrosine phosphatase alpha (PTPα) binds to integrin β1 subunit and can also activate Fyn and phosphorylate Akt to promote myelination (Ly et al., 2018). Thus, Fyn can be considered an integrator of axon-OL signals that control myelination. The activity of Fyn is transient, and it declines in stably myelinating OLs, but we do not yet understand the mechanism of its downregulation.

### Other Examples Extracellular Mechanism Suggestive of Fine-Tuning Myelination

#### Mechanisms that inhibit myelination

Most initial OL-axon contacts appear to result in process retraction, with only a minority becoming stable contacts (Snaidero and Simons, 2017). Below we provide a brief description of cell surface factors that negatively impact myelination.

#### PSA (polysialic acid)-NCAM (neural cell adhesion molecule)

PSA is a long α2, 8-linked polymer of sialic acid. In the nervous system, it is most frequently attached to the extracellular portion of NCAM, another cell adhesion molecule related to L1 (Rutishauser and Landmesser, 1996). During development when axons navigate to their destination, they are fasciculated *via* adhesion molecules on the surface. The tight bundle facilitates their rapid phase of growth through a permissive terrain established by the pioneer axon. NCAM on these migrating axons does not carry PSA. Once the axons reach their destination, they must defasciculate to find their synaptic targets. In the target region, the PSA moiety is added to NCAM by the function of polysialyltransferases. The addition of PSA not only adds the bulk of the carbohydrate on NCAM, but the highly negative charge on PSA attracts water, which causes steric hindrance and weakens the homophilic cell adhesion of parallel axons *via* NCAM. PSA also inhibits the adhesion of other adhesion molecules such as L1. Beyond development, PSA is also implicated in synaptic plasticity and remodeling in the adult. Like L1, PSA-NCAM is downregulated concomitant with the onset of myelination (Charles et al., 2000). Removable of PSA with endoneuroaminidase N enhances myelination, though it is not clear whether this occurs by increased adhesion between the axon and the myelinating OL. Since PSA levels on developing axons appear to be influenced by synaptic activity (Rutishauser and Landmesser, 1991), PSA could be another possible mechanism for relaying the “readiness” of the axon to be myelinated.

#### LINGO1

LINGO1 (Leucine-rich repeat and immunoglobulin domain-containing-1), a transmembrane protein expressed in neurons and OL lineage cells, also acts to negatively regulate the onset of myelination through downstream activation of the small GTPase RhoA, which inhibits the morphological differentiation of OLs (Mi et al., 2005). Interestingly, it was shown that the extracellular domain of LINGO1 expressed by other cell types can act as its own ligand, binding to OL-expressed LINGO1 and inhibiting differentiation and myelination (Jepson et al., 2012). It was also shown that independently modulating the expression of LINGO1 in either neurons or OLs is sufficient to produce effects on differentiation and myelination (Lee et al., 2007). Therefore, it has been proposed that the inhibitory effect of LINGO1 on OL differentiation is mediated by the trans-interaction of LINGO1 at OL-axon contact sites (Jepson et al., 2012).

#### Arf6-mediated trafficking in neurons affects myelination

Studies on cell type-specific roles of ADP-ribosylation factor 6 (Arf6) provide an interesting example of how specific secretory mechanisms affect OL differentiation in a cell non-autonomous manner. Arf6 is the sole member of class III Arf gene products and is expressed by many cell types in the CNS. While Arfs 1–5 play a role in vesicular trafficking between the ER and Golgi compartments, Arf6 is localized to the plasma membrane and endosomal compartments and regulates endocytosis of the plasma membrane, endosomal recycling, and exocytosis. Arf6 conditional knockout using *Nestin*-cre results in severe hypomyelination specifically in the corpus callosum and hippocampal fimbria (Akiyama et al., 2014). Since *Nestin-cre* causes the deletion of Arf6 from both neurons and OL lineage cells, the authors further generated conditional Arf6 knockout mice in which Arf6 is deleted specifically from neurons or from OLs. Interestingly, hypomyelination is observed in neuronal Arf6 knockout mice but not in OL-specific Arf6 knockout mice. The defect appears to be in OPC migration due to a defect in neuronal secretion of FGF2, which could be rescued by exogenous FGF2 added to the neuronal compartment in a Boyden chamber migration assay with wt OPCs and Arf6-deleted neurons. This illustrates how specific trafficking mechanism in non-OL lineage cells can severely affect OL-mediated myelination in an axon tract-specific manner.

## Concluding Remarks

A large body of literature exists on how individual molecules or signaling pathways affect OL development and myelination. Much of the knowledge has been obtained by applying specific molecules to OL lineage cells in culture or manipulating them individually *in vivo* and analyzing the phenotype of the cells or mice as a consequence of such an all-or-none type of manipulation. However, we understand little about the mechanisms that integrate multiple signals arriving at OL lineage cells in various strengths and forms to produce the desired effect on myelination. The recent surge of interest in adaptive myelination has revealed that OLs are highly sensitive to subtle network changes, and that their intracellular program to generate myelin is dynamically modulated throughout adult life. To obtain an integrated understanding of how neuronal activity and other factors in their microenvironment influence OL dynamics, there is a need to reevaluate the mechanism of action of various oligodendrogliogenic and myelinogenic factors by taking into consideration the dynamic ways in which these factors are made available to OL lineage cells.

To illustrate the diverse ways in which extracellular signals can be delivered to OL lineage cells, we have provided examples in the soluble and integral membrane proteins, BDNF and L1, respectively, often drawing from their mechanism of action studied in neurons. Additionally, we have reviewed the evidence that suggests that the non-receptor intracellular tyrosine kinase Fyn serves as a converging node of multiple extracellular signals during myelination. An integrated view of how multiple factors dynamically activate appropriate signaling pathways in OLs to produce a specific effect will require not only identification of what the specific signals are but also a way to multidimensionally assess the temporo-spatial profiles of the signals and their volume/intensity. This will require new approaches to manipulating multiple variables in a physiologically relevant and graded manner and new quantitative approaches to detect the responses in OL lineage cells.

## Author Contributions

CF and AN wrote the manuscript. Both authors contributed to the article and approved the submitted version.

## Funding

This work was supported by National Institutes of Health (NIH; R01NS116182, PI: AN), SNARE complex-mediated exocytosis in oligodendrocyte differentiation and survival NIH (2R01NS073425, PI: AN), homeostatic regulation of NG2 cell dynamics.

## Conflict of Interest

The authors declare that the research was conducted in the absence of any commercial or financial relationships that could be construed as a potential conflict of interest.

## Publisher’s Note

All claims expressed in this article are solely those of the authors and do not necessarily represent those of their affiliated organizations, or those of the publisher, the editors and the reviewers. Any product that may be evaluated in this article, or claim that may be made by its manufacturer, is not guaranteed or endorsed by the publisher.
